# Moderating Role of Psychological Flexibility in the Relationship Between Intolerance of Uncertainty and Perceived Stress Among Women Undergoing in Vitro Fertilization Treatment

**DOI:** 10.1002/brb3.71436

**Published:** 2026-05-05

**Authors:** Fatemeh karimzadeh, Abbas Abdollahi, Soolmaz Dehghanidowlatabadi

**Affiliations:** ^1^ Department of Counseling Faculty of Education and Psychology Alzahra University Tehran Iran; ^2^ Wellbeing Research Center UCSI University Kuala Lumpur Malaysia; ^3^ Faculty of Social Sciences and Liberal Arts UCSI University Kuala Lumpur Malaysia; ^4^ Department of Counseling, SR.C. Islamic Azad University Tehran Iran

**Keywords:** in vitro fertilization, intolerance of uncertainty, perceived stress, psychological flexibility

## Abstract

**Purpose:**

The aim of this study was to examine the moderating role of psychological flexibility (PF) in the relationship between intolerance of uncertainty (IU) and perceived stress (PS) among women undergoing in vitro fertilization (IVF) treatment.

**Methods:**

This study used purposive sampling and included 204 participants. The measurement instruments were the Intolerance of Ambiguity Scale (IUS‐12) developed by Carleton and colleagues (2007) for assessing IU, the Perceived Stress Scale (PSS‐10) developed by Cohen and colleagues (1983) for evaluating PS, and the Multidimensional Psychological Flexibility Inventory (MPFI‐60) developed by Rolffs and colleagues (2018) for assessing PF. A covariance‐based structural equation modeling using AMOS (version 24) software to test the direct relationships and multi‐group analysis was conducted to assess moderation effects of PF.

**Finding:**

The findings indicated that IU and Psychological Inflexibility (PI) positively predicted PS (β = 0.16, *p* < 0.001), while PF negatively predicted it (β = ‐0.14, *p* < 0.001). Multi‐group analysis revealed that in the low PF group, the association between IU and PS was significant, positive, and greater (β = 0.28, *p* < 0.001) than in the high PF group (β = 0.06, *p* = 0.13).

**Conclusion:**

Both variables, namely IU and PI, contribute to increased PS; however, PF moderates this relationship, indicating a protective role of PF in reducing PS among women undergoing IVF treatment.

## Graphical abstract

Intolerance of uncertainty (IU) and psychological inflexibility (PI) positively predict perceived stress (PS) among women undergoing IVF treatment. Psychological flexibility (PF) moderates this relationship, reducing the effect of IU on PS. Women with higher PF experience lower PS despite uncertainty, highlighting the protective role of PF in stressful reproductive contexts.

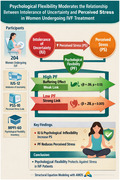

## 1 Introduction

In vitro fertilization (IVF) treatment is widely recognized as a psychologically demanding medical process for patients undergoing IVF, largely due to its invasive procedures, uncertain outcomes, and prolonged treatment trajectory (Eugster and Vingerhoets 1999). Quantitative evidence suggests that individuals undergoing IVF report significantly elevated stress levels compared to population norms, with mean scores on the perceived stress scale (PSS) ranging from approximately 18 to 22, exceeding commonly reported normative means of 12–14 (Eugster and Vingerhoets 1999). Psychological distress is further exacerbated by the substantial financial and temporal investment required for IVF, as patients often undergo multiple treatment cycles with no guarantee of success. Recent studies indicate that treatment‐related financial strain and repeated IVF attempts are significantly associated with higher perceived stress and emotional burden, accounting for approximately 25%–35% of the variance in stress‐related outcomes (Assaysh‐Öberg et al. 2023). Moreover, recent findings demonstrate that women undergoing assisted reproductive treatments report significantly higher stress and anxiety levels than comparison groups, with mean differences corresponding to moderate‐to‐large effect sizes (Cohen's d ≈ 0.50–0.80) (Aimagambetova et al. 2024; Fernandez‐Ferrera et al. 2022). Finally, previous research has shown that fear of treatment failure and perceived familial expectations are among the strongest predictors of psychological distress in IVF patients, jointly explaining approximately 30%–40% of the variance in stress and pressure symptoms (Aimagambetova et al. 2020; Assaysh‐Öberg et al. 2023).

One of the psychological factors that impacts the result of infertility treatments is intolerance of uncertainty (IU). IU is defined as a cognitive pattern primarily characterized by difficulties in dealing with unclear or ambiguous situations, which negatively affects an individual's cognitive, emotional, and behavioral processes. Individuals who exhibit higher levels of IU typically experience discomfort when facing uncertain circumstances and tend to actively avoid them; such behavior may hinder effective functioning and adaptation in situations with unclear outcomes (Rafieian et al. 2024). This cognitive bias shapes the way women undergoing IVF perceive, interpret, and react to stressful situations (Mousavi et al. 2020). Recent studies have confirmed that IU significantly affects PS, revealing that individuals with difficulty tolerating uncertainty generally experience higher levels of PS (Li and Song 2024; Sang et al. 2024). People with a high IU may perceive their disorders as significant threats and an indication of not reaching their goals. This misperception of stressors ultimately contributes to the persistence and escalation of PS (Xu et al. 2023). Although earlier studies have explored the link between IU and PS in situations like COVID‐19 (Palma et al. 2022), ego depletion (Li and Song 2024), and eating disorders (Konstantellou et al. 2019), this relationship has not been adequately investigated in women receiving IVF treatment. Therefore, this study attempts to investigate these relationships among women receiving IVF treatment.

To provide a clear theoretical framework, this study draws on the “stress and coping theory” (Lazarus and Folkman 1984), which explains the interaction between stressors and an individual's coping resources. According to this model, IU as a cognitive factor impairs an individual's ability to effectively cope with uncertain situations and increases PS. Previous research has shown that IU is associated with increased worry, psychological distress, and reduced coping performance (Carleton 2016). On the other hand, PF could act as a potential coping resource, allowing individuals to pursue meaningful goals and values rather than focusing on uncertainty, which may moderate the effect of IU on PS (Levin et al. 2016). This theoretical framework provides a solid logical basis for the research hypothesis that IU is related to PS and that PF plays a moderating role in this relationship.

Scientific literature highlights PF as a crucial protective factor for coping with uncertain situations (Abdollahi et al. 2022; Okayama et al. 2024). Additionally, research has validated the hypothesis that there is a connection between PF and IU (Daşcı et al. 2023). PF likely acts as a mental health safeguard for people with a high IU. It encourages them to pursue meaningful goals and values in life, rather than becoming fixated on uncertainty (Okayama et al. 2024).

In addition to the theoretical framework, the timing of data collection is crucial for an accurate interpretation of the effect of IU and PF on PS in women undergoing IVF. In this study, questionnaires were completed at the “post‐ovarian stimulation and pre‐embryo transfer” stage, as this stage is considered the most sensitive point for measuring PS due to the psychological burden of uncertainty in treatment outcomes, concerns about ovarian response, financial and social consequences, and anxiety related to the treatment process (Abdollahi et al. 2021; Gameiro et al. 2015). Studies showed that stress levels could vary during the IVF cycle and are significantly different from the embryo transfer stage in the early stages or after a failed cycle (Boivin et al. 2011). Therefore, clarifying the data collection stage allows for a more accurate assessment of the effect of IU and the moderating role of PF and helps in the interpretation of the results.

In a previous study, PF moderated the relationship between IU and psychological distress/physical symptoms (O'Brien et al. 2021). The impact of PF in moderating IU during the COVID‐19 pandemic has been studied (Mallett et al. 2021; O'Brien et al. 2021), but there is a lack of research on how PF moderates the relationship between IU and PS in women undergoing IVF treatment. The outcome of treatment, financial burden, social consequences of infertility, and fear of pain associated with treatment methods lead to stress in women undergoing IVF. However, this pressure may be alleviated with flexibility throughout the treatment process (Patel et al. 2024). Recent findings in various communities indicate a negative relationship between flexibility and greater levels of stress in individuals (Deng et al. 2023; Holding et al. 2024; Puolakanaho et al. 2023).

Since PF allows individuals to fully accept personal life events, it may help reduce their stress levels (Arbinaga Ibarzabal et al. 2024). PF may also act as a moderator between negative events and stress (Baker et al. 2022). In the Hexaflex model of Acceptance and Commitment Therapy (ACT), six core processes are identified to enhance PF: (1) Acceptance: The ability to embrace painful thoughts and feelings rather than attempting to control or suppress them. (2) Diffusion: The capacity to observe and notice negative thoughts and feelings without interpreting them as absolute truths or becoming entangled in them. (3) Present‐moment mindfulness: Full, aware engagement in the here and now, free from distraction and judgment. (4) Self‐as‐context: Maintaining a flexible perspective on oneself, independent of thoughts and feelings, and perceiving oneself as a consistent observer. (5) Values‐based committed contact: The ability to connect deeply and openly with one's personal goals, meaning, and values without resistance or avoidance. (6) Committed action: Purposeful and goal‐oriented behaviors aligned with personal values, even when faced with significant difficulties (Azadfar et al. 2022; Hayes et al. 2012; Hayes and Pierson 2005).

While previous studies have examined the moderating role of PF in relation to mental health and stress in cases of trauma (Boykin et al. 2020), COVID‐19 (Yao et al. 2023), eating disorders (Ferreira et al. 2011), and breast cancer (Novakov 2021), no study has conducted the moderating role of PF in women undergoing IVF treatment. Therefore, it may be hypothesized that there is a positive relationship between IU and PS, and PF would moderate the relationship between IU and PS.

## 2 Method

### 2.1 Participants

A total of 204 women undergoing IVF treatment, ranging in age from 27 to 48 years (*M*  =  45.32  ±  8.2), participated in the present study. Regarding education, 28 participants (13.7%) were under diploma, 56 participants (27.5%) had a diploma, 77 participants (37.7%) held a bachelor's degree, 37 participants (18.1%) held a master's degree, and 9 (4.4%) participants held a doctoral degree (See Table 1). Table 1 also reports means ± standard deviations of continuous variables including age, IU, PF, PI, and PS. Education levels, as a categorical variable, are presented as frequency and percentage in five educational groups (less than diploma, diploma, bachelor's degree, master's degree, and doctorate). Given that PS was the dependent variable of the study, the difference in mean scores of PS across different education levels was examined using a one‐way ANOVA test, and the *p*‐value for this comparison is reported in the table. The results of ANOVA showed that there were statistically significant differences between education levels in terms of PS (*p* = 0.031). However, the interpretation of this finding requires caution. The distribution of sample sizes across educational groups was unbalanced, and in particular, the number of participants with a PhD was relatively limited. This imbalance could increase the sensitivity of the ANOVA test to mean differences, without necessarily indicating stable and generalizable differences in the target population. Therefore, although the results support the existence of a statistical difference, this difference may be partially affected by the small sizes of groups.

**TABLE 1:** Demographic characteristics and descriptive statistics of key variables (*N* = 204).

Variable	Category	n	%	Mean ± SD	Perceived stress (Mean ± SD)	*p*‐value
Age (years)	—	—	—	45.32 ± 8.20	—	—
Education level	Below diploma	28	13.7	—	27.1 ± 6.9	
	Diploma	56	27.5	—	25.6 ± 6.7	
	Bachelor's	77	37.7	—	23.9 ± 6.5	
	Master's	37	18.1	—	22.4 ± 6.2	
	Doctorate	9	4.4	—	21.8 ± 5.9	0.031^1^
Intolerance of uncertainty	—	—	—	68.40 ± 12.50	—	—
Psychological flexibility	—	—	—	72.10 ± 10.30	—	—
Psychological inflexibility	—	—	—	58.70 ± 11.20	—	—
Perceived stress	—	—	—	24.50 ± 6.80	—	—

^1^ One‐way ANOVA comparing perceived stress across education levels.

### 2.2 Procedure

The Iranian Ministry of Health, as well as heads of three hospitals in Tehran, gave permission to collect data. The duration of data collection is for a month and a half, starting from April 30, 2023. All participants had already received IVF treatment from the specialists in the hospital, and the researchers gave their email addresses and phone numbers from the hospital. The questionnaires were placed on the Porsline platform (Iranian website), and the participant's consent form was placed on the starting page of the online questionnaire. The participants signed the written informed consent forms before completing the questionnaires. The link to the questionnaires was provided to the participants through email, Telegram, WhatsApp, and ETA social networks.

Given the average completion time of approximately 45 min, which could lead to survey fatigue, decreased attention, inaccurate responses, and dropout, this study employed methodological strategies to mitigate these effects. Studies have shown that longer questionnaires are associated with increased likelihood of incomplete responses, decreased response quality, and increased dropout rates, with each hour of additional response time significantly increasing the rate of question omission (Ying et al. 2016). Length also reduces participant satisfaction and attention, which may lead to systematic bias in the data (Berry et al. 2022).

To reduce these effects and ensure data quality, the following measures were taken: (1) the questionnaires were presented in a modular format to reduce the cognitive load of the respondents and reduce the feeling of fatigue during the response process, (2) the answers in each section were automatically saved so that the respondent could complete the response in several sessions, (3) and the questionnaire design used a “progress bar” and clear explanations about the approximate completion time to manage participants’ expectations to maintain participants’ motivation. These methods are used to reduce self‐selection bias, maintain motivation, and improve response accuracy in online studies.

### 2.3 Measures

#### 2.3.1 Intolerance of Uncertainty Scale‐12 (IUS‐12)

The IUS‐12 scale was employed to assess IU. This measure consists of 12 items, such as “ unforeseen events upset me greatly,” and utilizes a 5‐point Likert scale from 1 “Not at all characteristic of me” to 5 “Very Characteristic of me”. A previous study reported an acceptable internal consistency with a Cronbach's Alpha of 0.96 (Carleton et al. 2007). In this study, the Cronbach's alpha for the IUS‐12 was found to be 0.87. In addition to internal consistency reliability, the construct validity of the scale was also examined in the present sample. As shown in Table 2, both composite reliability (CR = 0.71) and average variance extracted (AVE = 0.60) exceeded the proposed threshold values (CR > 0.70 and AVE > 0.50) (Hair et al. 2014), indicating adequate convergent validity and acceptable construct validity of the IUS‐12 in this study.

**TABLE 2:** Values of construct reliability, average variance extracted.

Variable	CR^1^	AVE^2^
Perceived stress	0.72	0.61
Intolerance of uncertainty	0.71	0.60
Psychological flexibility	0.73	0.58
Psychological inflexibility	0.72	0.59

^1^ Construct reliability.

^2^ Average variance extracted.

#### 2.3.2 Perceived Stress Scale‐10 (PSS‐10)

PS was measured using the shortened “Perceived Stress Scale” (Cohen et al. 1983). The PSS‐10 evaluates PS levels over the past month and consists of 10 Likert‐type items (e.g., “In the last month, how often have you felt unable to handle all the things you had to do?”) rated from 0 “never” to 4 “very often.” The total score ranges from 0 to 40, with higher scores indicating greater levels of PS. Items 6, 7, 8, and 9 are reverse‐scored. Based on the current sample, the convergent validity (AVE) was 0.68, and the Cronbach's alpha was 0.83. In this study, the Cronbach's alpha for the PSS‐10 was found to be 0.86. In order to assess the validity, convergent validity indices were calculated. According to Table 2, the composite reliability value (CR = 0.72) and the average variance extracted (AVE = 0.61) for the PSS‐10 were within the acceptable range, indicating the adequacy of the convergent validity and construct validity of this instrument in the present study sample.

#### 2.3.3 Psychological Flexibility Scale (MPFI‐60)

PF was measured using the Multidimensional Psychological Flexibility Inventory (MPFI‐60) (Rolffs et al. 2018). The MPFI is a 60‐item measure used to assess both PF and PI. The PF dimension includes six subscales—acceptance, present‐moment awareness, self as context, diffusion, values, and committed action—with each subscale represented by 5 items. Conversely, the PI dimension also features six subscales—experiential avoidance, lack of present moment contact, self as content, fusion, disconnection from values, and inaction—each with 5 items. Responses are rated from 1 (never) to 6 (always). Scores for each dimension are derived from the sum of the subscales, with higher scores reflecting greater levels of PF or PI (Rolffs et al. 2018). The Cronbach's alpha coefficient for PF in this study was calculated as 0.95, and for PI, it was 0.93. In addition to reliability, the construct validity of both dimensions of the MPFI‐60 was also examined. As reported in Table 2, the composite reliability for PF (CR = 0.73) and PI (CR = 0.72) was at acceptable levels. Also, the AVE values for PF (0.58) and PI (0.59) were above the threshold of 0.50, indicating adequate convergent validity and adequate measurement validity of the MPFI‐60 in the present study sample.

### 2.4 Statistical Method

A covariance‐based structural equation model with AMOS software (version 24) was used to address the research hypotheses in this study (Arbuckle 2006). Data is analyzed in three steps using structural equation modeling.

- The measurement model involved evaluating convergent validity and construct reliability (when AVE and CR values were greater than 0.5 and 0.7, respectively, indicating the measure has acceptable convergent validity and internal consistency (Bryne 2010)). The proposed model is fitted when measurement model fit indices meet the criteria (CMIN/df <5; Root Mean Squared Error of Approximation (RMSEA) <0.08; Tucker‐Lewis Index (TLI), Comparative Fit Index (CFI), and Goodness of Fit Index (GFI) >0.90).
- The structural model involved evaluating the coefficient of determination (R2) and the beta coefficients of the relationships between the outcome variable (PS) and the predicting variables (PF, PI, and IU).
- Moderation analysis involved examining whether the moderating variable (PF) alters the direction or intensity of the link between the predicting variable (IU) and outcome variables (PS).

### 2.5 Measurement Model

**FIGURE 1:**
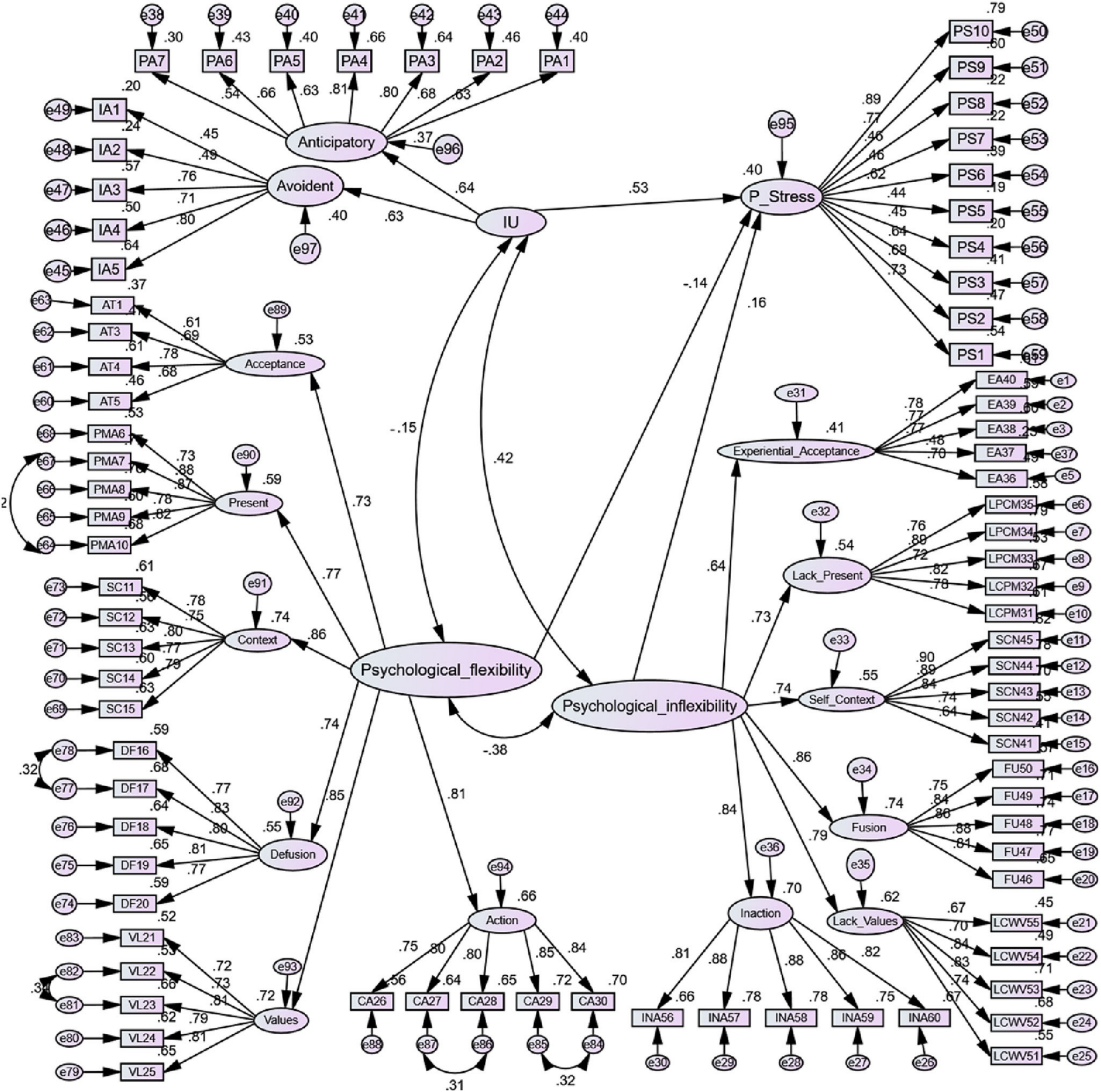
Structural model of perceived stress (*p* < 0.001).

Figure 1 shows that all standardized factor loadings were significant and greater than 0.50 and less than 1, indicating that the items loaded adequately on the respective constructs (Kline 2015). The fit indices of the measurement model showed that the model had a good fit with the data, as all indices were within the acceptable range (CMIN/df = 3.81, *p* < 0.01, CFI = 0.94, TLI = 0.94, RMSEA = 0.05, and GFI = 0.93).

### 2.6 Structural Model

After the measurement model was confirmed, a structural model was estimated to test the hypotheses. The full results of the structural paths including standardized coefficients (β), standard errors (SE), *p* values, and 95% confidence intervals are reported in Table 3. The results showed that IU positively and significantly predicted PS (β = 0.53, SE = 0.06, 95% CI [0.41, 0.65], *p* < 0.001). Also, PI had a positive and significant effect on PS (β = 0.16, SE = 0.05, 95% CI [0.07, 0.26], *p* < 0.001). In contrast, PF significantly and negatively predicted PS (β = −0.14, SE = 0.05, 95% CI [−0.24, −0.05], *p* < 0.001).

**TABLE 3:** Structural paths, standard errors, and 95% confidence intervals.

Outcome variable	Predictor variable	Standardized β	SE	95% CI	*p*‐value
Perceived stress	Intolerance of uncertainty	0.53	0.06	0.41 – 0.65	<0.001
Perceived stress	Psychological inflexibility	0.16	0.05	0.07 – 0.26	<0.001
Perceived stress	Psychological flexibility	−0.14	0.05	−0.24 – −0.05	<0.001

The coefficient of determination (R^2^) for PS was 0.40 (95% CI [0.33, 0.47]), indicating that IU, PF, and PI together explained 40% of the variance in PS (see Figure 1). This value exceeds the threshold of 0.35, which is considered a moderate to high value for the coefficient of determination in structural models (Henseler et al. 2015).

### 2.7 Moderation Analysis of PF

To examine the moderating role of PF in the relationship between IU and PS, a multigroup analysis was used within the CB‐SEM framework (Preacher and Hayes 2008). The median value of PF was 74, and the sample was divided into two groups: the low PF group (*n* = 100, 49%) the and high PF group (*n* = 104, 51%). To test the moderating effect, the invariant group (restricted) and variant group (unrestricted) models were compared. The judgment criterion was the improvement in the fit indices of the variant group model compared to the invariant group model (Byrne 2013). The results showed that the fit indices of the variant group model (χ^2^ = 2.72, *p* < 0.01, RMSEA = 0.07, CFI = 0.91, GFI = 0.90, NFI = 0.91) were significantly better than the invariant group model (χ^2^ = 5.57, *p* < 0.01, RMSEA = 0.09, CFI = 0.75, GFI = 0.74, NFI = 0.73), indicating the moderating effect of PF.

The results of multi‐group analysis showed that in the low PF group, the relationship between IU and PS was positive and significant (β = 0.28, SE = 0.07, 95% CI [0.14, 0.42], *p* < 0.001), while in the high PF group this relationship was not significant (β = 0.06, SE = 0.05, 95% CI [−0.04, 0.16], *p* = 0.13). This finding suggests that PF attenuates the magnitude of the effect of IU on PS.

### 2.8 Moderation Analysis of PI

To test the moderating role of PI in the relationship between IU and PS, a multi‐group analysis was employed (Preacher and Hayes 2008). The median value of PI was 65, and the sample size was divided based on the median value into two groups (the low PI group with 125 (61%) participants and the high PI group with 79 (39%) participants).

The results showed that the invariant group model's fit indices (χ2 = 4.57, *p* < 0.01, RMSEA = 0.81, CFI = 0.83, GFI = 0.84, NFI = 0.83) had better measurement fit indices than the fit index for the variant group model's fit indices (χ2 = 5.57, *p* < 0.01, RMSEA = 0.073, CFI = 0.75, GFI = 0.74, NFI = 0.73), indicating the lack of a moderating role of the PI variable in the relationship between IU and PS.

## 3 Discussion

This study aimed to investigate the moderating role of PF in the relationship between IU and PS among women undergoing IVF treatment. Data from 204 women admitted to a specialized infertility center were analyzed using structural equation modeling. The results showed that IU and PI were positively associated with increased PS, while PF was inversely associated with PS. To test for moderation, a multigroup SEM analysis was conducted based on low and high levels of PF. The findings showed that the relationship between IU and PS was significantly stronger in the low PF group than in the high PF group, suggesting a contextual moderating role of PF in this relationship.

The finding of this study stated that IU is positively and significantly related to PS in women undergoing IVF treatment. This finding is consistent with a body of previous research in the field of infertility, mental health, and medical conditions associated with uncertainty (Abdollahi and Abu Talib 2015; Assaysh‐Öberg et al. 2023; Li and Song 2024; Mousavi et al. 2020; Sang et al. 2024). Previous research has shown that fertility treatments, especially IVF, are structurally associated with high levels of uncertainty; uncertainty about the outcome of treatment, duration, number of cycles, and physical and financial consequences have all been reported as persistent sources of stress (Mousavi et al. 2020). Consistent studies emphasize that individuals with high IU not only experience anxiety more frequently but also experience greater intensity and persistence of stress because they perceive ambiguity as inherently intolerable and threatening. This finding could be explained within the framework of Lazarus and Folkman's theory of PS (Lazarus and Folkman 1984). According to this theory, stress is not a direct consequence of external events but rather the result of cognitive appraisal processes. In the initial appraisal phase, individuals with high intolerance of ambiguity evaluate the IVF situation as a serious threat to vital goals (such as motherhood, female identity, and future security). In the secondary appraisal phase, these individuals also perceive their coping resources as inadequate and feel little control over the consequences. This dual appraisal pattern causes uncertainty to be experienced not only as a situational feature, but also as a persistent psychological strain, ultimately leading to increased PS.

The finding of this study predicted that PF would have a negative and significant relationship with PS. The findings of this study indicated that women with higher levels of PF would experience less PS. This result is consistent with a significant body of previous research that has identified PF as a protective factor against stress, anxiety, and depression (Baker et al. 2022; Deng et al. 2023; Puolakanaho et al. 2023). Consistent studies suggest that in challenging medical situations, individuals with high PF exhibit milder and more adaptive emotional responses despite facing the same level of objective stress. From the perspective of the six‐pronged model of ACT (Hayes, Strosahl, et al. 2012), this negative relationship is explained by the simultaneous operation of six key processes. Acceptance could prevent the destructive engagement with negative emotions and may allow anxiety to be experienced without escalation. Cognitive decoupling could reduce the power of threatening thoughts about the outcome of treatment and prevent the individual from becoming absorbed in catastrophic scenarios. Contact with the present moment could divert attention from future‐oriented concerns and conserve cognitive resources. The self as a context could prevent the generalization of treatment failure or success to personal worth. Clarity of values could provide a stable semantic framework, and committed action reinforces a sense of continued agency and efficacy (Abdollahi et al. 2020; Fattahi et al. 2023). Collectively, these processes make the experience of uncertainty less likely to translate into PS.

The finding of this study also stated that PI is positively and significantly related to PS. The findings of the present study assessed this hypothesis and are consistent with previous studies that have identified strong predictors of PI (Baker et al. 2022; Deng et al. 2023; Puolakanaho et al. 2023). Consistent studies show that in clinical populations, PI not only increases stress but also could prevent it from decreasing over time. According to the ACT perspective (Hayes, Strosahl, et al. 2012), PI results from the dominance of the six‐sided maladaptive process. Experiences are transformed into persistent attempts to eliminate unpleasant emotions, which themselves exacerbate stress. Cognitive fusion could turn negative thoughts into undeniable truth. Disengagement from the present moment could keep the individual in continuous rumination. The conceptualized self‐causes the individual's identity to be tied to the outcome of the treatment. The lack of clear values could create the experience of meaninglessness, and behavioral passivity reinforces feelings of helplessness (Howell and Passmore 2019).

The results of the multigroup analysis showed that in women with low PF, the relationship between IU and PS was stronger, while this relationship was weakened in the high flexibility group. This pattern is consistent with the findings of previous research showing that PF could reduce the effect of cognitive vulnerability factors (Arbinaga et al. 2024; Baker et al. 2022). From an ACT theoretical perspective (Hayes, Strosahl, et al. 2012), PF could act as a regulatory context that changes the path of uncertainty into stress. The presence of processes such as acceptance, cognitive dissonance, and value orientation could prevent the threat‐oriented appraisal caused by IU from directly leading to severe stress. Even if the interaction effect is not significant in the direct test, group differences suggest that PF plays a protective role by weakening the cognitive‐emotional link between IU and stress.

The results also showed that PI did not play a significant moderating role in this relationship. This finding is consistent with a previous study that showed that inflexibility could act more as a general risk factor than as a moderating variable (Deng et al. 2023). From an ACT perspective (Wersebe et al. 2018), this result could be explained because PI is a set of pervasive and stable processes that may lead to increased stress at almost all levels of IU. When experiential avoidance, cognitive fusion, and value dissociation are dominant, IU is more likely to lead to high stress, and the space for variability necessary for the formation of a moderating effect is lost. Therefore, PI does not change the direction of the IU–stress relationship but rather exacerbates it overall.

### 3.1 Theoretical Contribution

By combining Folkman and Lazarus' (1984) PS theory and the six‐dimensional framework of ACT, this study suggests an integrated theoretical framework to examine the relationships between IU, PF, and PS. According to PS theory (Lazarus and Folkman 1984), stress is experienced when an individual lacks adequate coping resources to respond to environmental threats. On the other hand, PF includes six key dimensions, including acceptance, presence in the moment, self as context, values, committed action, and cognitive flexibility, that enhance an individual's ability to manage ambiguous experiences. The findings of this study suggest that PF could act as a contextual coping resource, such that women with high PF experience less of the effect of IU on stress, while PI involves the lack of any of these six skills, leading to increased PS. This theoretical framework helps to clarify the cognitive‐emotional mechanisms involved in dealing with uncertainty in clinical settings and outlines future research directions for the development of targeted preventions.

### 3.2 Practical Contribution

Given the ambiguous nature of the IVF treatment process, the findings of this study suggest that identifying women with high IU and low PF could allow for the design of targeted psychological support and preventive programs. Although the findings of this study are not interventional, it is conceivable that preventive programs based on increasing PF, especially strengthening the skills of acceptance, presence in the moment, and committed action, could improve the ability to manage ambiguity and reduce PS.

### 3.3 Future Recommendations and Limitations

This study had several limitations that should be considered in interpreting the findings. First, the reliance on self‐report questionnaires limits the possibility of causal inference; future studies could increase the validity of the results by using multimethod approaches, including empirical or longitudinal assessments. Second, the study sample was limited to women undergoing IVF treatment; therefore, generalization of the results to other populations requires caution, and future studies could reproduce the present model in diverse clinical and general groups. Third, although the sample size was sufficient for SEM analyses, replication of the study with larger and more diverse samples could strengthen external validity. Fourth, this study mainly focused on the moderating role of PF; examining other potential moderating factors, such as resilience or hardiness, could provide more detailed explanations and a more comprehensive theoretical framework.

## 4 Conclusion

The present study showed that IU and PI are positively associated with PS in women undergoing IVF treatment. The findings also showed that PF had a moderating role, such that the effect of IU on PS was reduced in women with high PF compared to women with low PF. These results suggest that PF could act as a psychological protective factor against the negative effects of IU on PS.

## Author Contributions

**Fatemeh Karimzadeh** and **Abbas Abdollahi** contributed to writing the manuscript, conducted data analysis, and interpreted the findings according to content areas, reaching an agreement on the classifications. **Soolmaz Dehghanidowlatabadi** provided supervision and revised the manuscript. All authors have read and approved the final version of the manuscript.

## Funding

The authors have nothing to report.

## Ethics Statement

The study was reviewed and approved by the Ethics Committee of Alzahra University (Approval No. 1402/PSY/15). All procedures were conducted in accordance with the ethical standards of the institutional research committee and the 1964 Helsinki Declaration and its later amendments. Written informed consent was obtained from all participants prior to participation.

## Consent

Informed consent was obtained from all individual participants in the study.

## Conflicts of Interest

The authors declare no conflicts of interest.

## Data Availability

Data is available on the Figshare repository at https://doi.org/10.6084/m9.figshare.31896526.
